# Resveratrol protects against *Schistosoma mansoni-*induced liver fibrosis by targeting the Sirt-1/NF-κB axis

**DOI:** 10.1007/s10787-023-01382-y

**Published:** 2023-12-02

**Authors:** Dalia Kamal Mostafa, Maha M. Eissa, Doaa A. Ghareeb, Shaymaa Abdulmalek, Wafaa A. Hewedy

**Affiliations:** 1 Clinical Pharmacology Department, Faculty of Medicine, Alexandria University, Al-Moassat Medical Campus, Elhadara, Alexandria, 21561 Egypt; 2 Medical Parasitology Department, Faculty of Medicine, Alexandria University, Alexandria, Egypt; 3 Biochemistry Department, Faculty of Science, Alexandria University, Alexandria, Egypt

**Keywords:** Liver fibrosis, NF-κB, Portal pressure, Resveratrol, Schistosomiasis

## Abstract

Hepatic schistosomiasis is a prevalent form of chronic liver disease that drastically affects human health. Nevertheless, an antifibrotic drug that could suppress the development of hepatic fibrosis does not exist yet. The current study aimed to evaluate the effect of resveratrol, a natural polyphenol with multiple biological activities, on *Schistosoma mansoni* (*S. mansoni*)-induced hepatic fibrosis and delineate the underlying molecular mechanism. Swiss male albino mice were randomly assigned into infected and non-infected groups. Hepatic schistosomiasis infection was induced via exposure to *S. mansoni* cercariae. 6 weeks later, resveratrol was administrated either as 20 mg/kg/day or 100 mg/kg/day for 4 weeks to two infected groups. Another group received vehicle and served as infected control group. At the end of the study, portal hemodynamic, biochemical, and histopathological evaluation of liver tissues were conducted. Remarkably, resveratrol significantly reduced portal pressure, portal and mesenteric flow in a dose-dependent manner. It improved several key features of hepatic injury as evidenced biochemically by a significant reduction of bilirubin and liver enzymes, and histologically by amelioration of the granulomatous and inflammatory reactions. In line, resveratrol reduced the expression of pro-inflammatory markers; TNF-α, IL-1β and MCP-1 mRNA, together with fibrotic markers; collagen-1, TGF-β1 and α-SMA. Moreover, resveratrol restored SIRT1/NF-κB balance in hepatic tissues which is the main switch-off control for all the fibrotic and inflammatory mechanisms. Taken together, it can be inferred that resveratrol possesses a possible anti-fibrotic effect that can halt the progression of hepatic schistosomiasis via targeting SIRT1/ NF-κB signaling.

## Introduction

Schistosomiasis is a devastating neglected tropical disease commonly affecting people in low-income developing countries. It is a serious public health problem that significantly impacts morbidity, mortality, and socioeconomic status of the affected population (Wilson et al. 2019). Hepatic granuloma caused by S*. mansoni* is a pathognomonic feature of hepatic schistosomiasis. It continues to be one of the most prevalent causes of chronic liver disease worldwide. Hepatic schistosomiasis arises from a destructive inflammatory reaction triggered by the host immune system towards the parasite eggs trapped in the liver (Eissa et al. 2020). Soluble egg-antigens (SEA) secreted primarily by the mature eggs elicit a type-2 inflammatory response that induces peri-ovular granuloma formation. On one hand, the formed granulomas play a beneficial role to the host where they guard against the direct exposure to egg antigens. Nevertheless, the unregulated inflammatory response to untreated granulomas is a crucial etiopathological factor in the development of hepatic fibrosis by *S. mansoni* infection (Eissa et al. 2020; Hams et al. 2013). Hepatosplenomegaly, portal hypertension, and life-threatening variceal bleeding are serious sequelae of irreversible hepatic fibrosis as they may be potentially fatal in the absence of medical attention. Thus, interruption of this vicious circle is critically warranted especially with lack of an effective therapy that can reverse fibrosis.

During periovular granuloma formation, SEA induces a Th2 response which is primarily orchestrated by CD4 + T cells. The nuclear factor kappa B (NF-κB) family is crucial for the development of the subsequent inflammatory response (Muriel 2009). Under physiological conditions, NF-κB is retained dormant in the cytoplasm via binding to its inhibitory proteins, Iκβs (O’Dea et al. 2007). Upon activation, NF-κB translocates to the nucleus to regulate a variety of inflammatory genes, particularly TNF-α and IL-1β, within various immune cells. The cross talk between these cytokines and chemokines triggers the activation of hepatic stellate cells that amplify the inflammatory response and promote granuloma formation (Liu et al. 2017; Zhangdi et al. 2019).

At the initial stage of schistosomiasis, management focuses on the eradication of infection and prevention of the development of hepatic fibrosis. Whilst after the development of the severe form of the disease, management of complications of portal hypertension is the main target. Praziquantel (PZQ) is the main strategy for parasite elimination at current. However, the effectiveness of PZQ on hepatic lesions is very minimal and is ascribed mainly to parasite eradication (Liang et al. 2011). This means that the likelihood of progression to hepatic fibrosis and cirrhosis still exists. Thus, to ensure liver protection against fibrosis and its subsequent complications, a combination of other (anti-fibrotic) drugs plus PZQ could be more advantageous than PZQ monotherapy (Fang et al. 2021). Therefore, promising plant derived compounds like resveratrol with long history of safety merits attention.

Resveratrol (3,5,4′trihydroxy-trans-stilbene) is a naturally occurring non-flavonoid polyphenol derived from various plant sources (Koushki et al. 2018). Owing to its pleiotropic pharmacological activities, resveratrol gains attention as a promising therapeutic agent in different pathological conditions (Malaguarnera 2019). Specifically, resveratrol shows promising results in different experimental models of liver diseases including chemical-induced hepatotoxicity, alcoholic and nonalcoholic fatty liver diseases, and hepatocellular carcinoma (Bishayee et al. 2010; Izzo et al. 2021).

Several studies revealed that the preventive and/or therapeutic effects of resveratrol are ascribed to its modulatory actions on a broad spectrum of molecular targets. For instance, resveratrol can reduce inflammatory markers through modulation of their transcription factors and their upstream signaling molecules (de Sá Coutinho et al. 2018). The potent anti-inflammatory effect of resveratrol was documented in various inflammatory conditions (Singh et al. 2019) Additionally, Resveratrol is described as a potent silent information regulator 1 (SIRT1) agonist (Iside et al. 2020). As a member of histone deacetylases (HDACs), SIRT1 enhances the deacetylation of regulatory molecules implicated in the cellular response to various conditions (Nogueiras et al. 2012). Mounting evidence sheds light on the critical role of deranged expression and/or activities of SIRT1 in various metabolic, neurologic, and cardiovascular diseases through epigenetic modifications of target proteins (Iside et al. 2020; Zhou et al. 2021). This notion has attracted the attention of many researchers to search for agents that can regulate SIRT1 activity.

Although previous studies demonstrated the ameliorative effect of resveratrol in animal models of schistosomiasis (Chen et al. 2019; El-Agamy et al. 2011; Soliman et al. 2017), none reported its effect on portal pressure. Additionally, the detailed molecular mechanism remains to be fully elucidated. In the present study, we hypothesized a link between the effect of resveratrol on the NF-κB/SIRT1 signaling pathway and slowing or suppressing the progression of hepatic schistosomiasis in a murine model of *S. mansoni* infection.

## Materials and methods

### Animals

The study was conducted on 48 Swiss male albino mice of CD-1 strain (20–25 g) purchased form the animal house of the department of Medical Parasitology, Faculty of Medicine, Alexandria University. Mice were maintained under standard laboratory conditions with access to food and water ad libitum. All experimental work was carried out according to NIH guide for use and care of laboratory animals where every effort was made to minimize animal suffering. The experimental protocol was approved by the local ethics committee at the Faculty of Medicine, Alexandria university, (Permit number: 0305944).

### Experimental design

**Fig.1:**
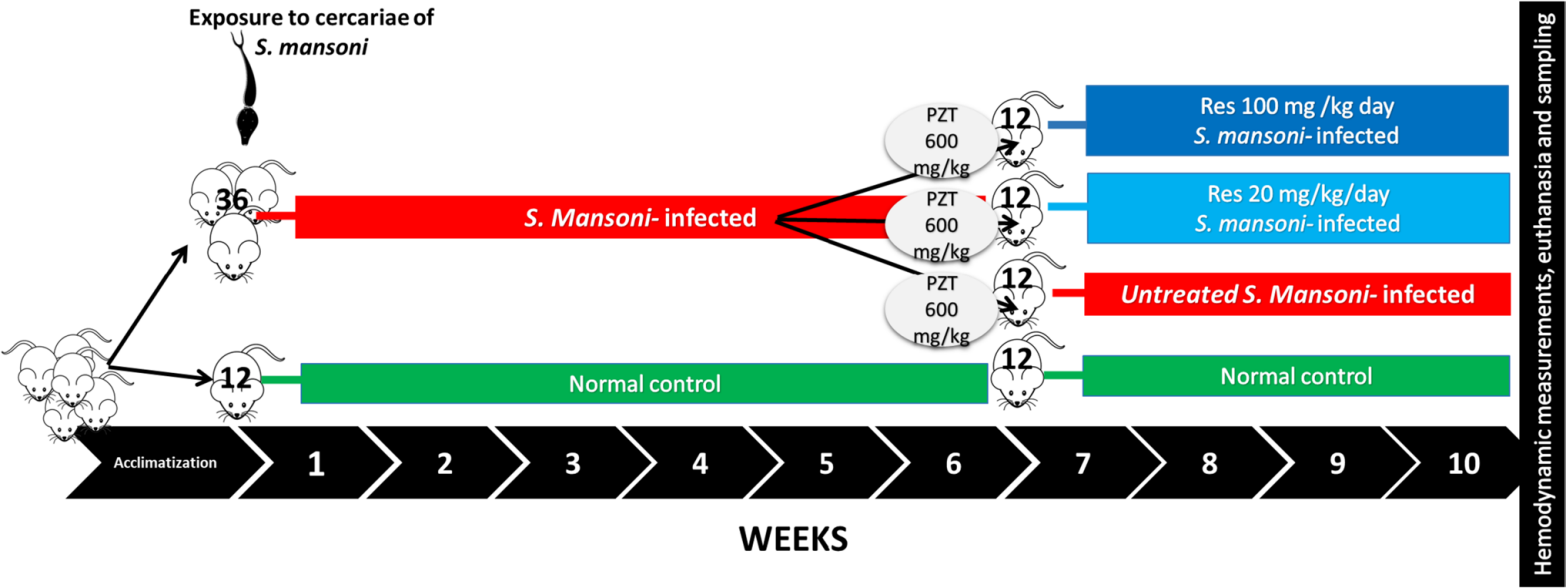
Schematic presentation of the timeline for the animal grouping and drug intake during the study. Res; resveratrol, PZQ; praziquantel

After 1 week of acclimatization, mice were randomly assigned to be either normal control (12 mice) or *S.* mansoni-infected (36 mice). 6 weeks following exposure to cercariae, infected animals received PZQ (600 mg/kg) once orally to eliminate adult worms (Liu et al. 2014) and then were further divided equally into 3 groups namely resveratrol-untreated S. mansoni-infected that received drug vehicle (For simplicity it will be referred to here as untreated infected group), and two resveratrol-treated groups receiving either 20 mg/kg (Res 20-infected) or 100 mg/kg (Res 100-infected) of resveratrol daily (Di Pascoli et al. 2013; Schwingel et al. 2014). Mice were sacrificed after 4 weeks of starting the treatments. Drugs were prepared in 2% gum acacia and were given by oral gavage. The normal control received gum acacia as a vehicle. A schematic presentation of the experimental design is shown in Fig. 1.

At the end of the study, blood was collected form the abdominal aorta for serum biochemical analysis. Animals were then euthanized, and the spleen and liver were carefully isolated, and their weights were determined. The liver of each animal was then cut into 2 halves to be either immediately frozen at – 80 ℃ or kept in 10% buffered formalin for biochemical and histopathological evaluation, respectively.

### Experimental procedures

#### Establishment of S. mansoni infection

The life cycle of Egyptian strain of Schistosoma *mansoni* was maintained in the Medical Parasitology Department, Faculty of Medicine, Alexandria University, by serial passages in laboratory bred *Biomphalaria alexandrina* snails and Swiss albino mice. Cercariae were shed from infected snails 25–30 days after exposure to miracidia. Each mouse of the infected group was infected with 100 ± 10 freshly shed cercariae using the paddling technique.

#### Hemodynamic studies

One day before the end of the study, each mouse was anaesthetized by i.p. ketamine 100 mg/kg and xylazine (10 mg/kg) for blood flow and pressure measurements for blood flow and pressure measurements (Wenger 2012). Briefly, a transverse abdominal incision was made, and the intestine was exteriorized to the left side and covered with a wet gauze. The superior mesenteric artery was localized and dissected, and a perivascular transonic flow probe (MA0.7PSB) connected to the transonic flow probe system-TS420 (Transonic systems^®^ Inc) and the Powerlab 8/35 (ADINSTRUMENTS) was placed around the artery. The mesenteric blood flow (MBF) was recorded for 5 min as mL/min using the labchart pro software (ADINSTRUMENTS ^®^) (Xie et al. 2014). The probe was then placed around the portal vein, as close as possible to the liver to avoid portal-collateral blood flow, for another 5 min to measure portal blood flow (PBF; as mL/min) going through the liver (Di Pascoli et al. 2013). To detect portal hypertension, a PE 10 catheter (Intradmedic Clay Adams1 non-radio-opaque polyethylene tubing, Becton Dickinson Primary Care Diagnostics) connected to a physiological pressure transducer (MLT844, AD Instruments) was inserted into the superior mesenteric vein and advanced into the portal vein for measuring the portal pressure (Geerts et al. 2008). Pressure signals were recorded by the power lab system (Power Lab 8/35 with Lab Chart Pro Module Software AD instrument. Australia.

#### Biochemical estimates

##### Serum biochemical assays

The collected blood was immediately centrifuged, and the serum was kept at -20 for further evaluation of liver enzyme functions. Commercial kits (Spectrum Diagnostics, Egypt) were used to assay Alanine transaminase (ALT), Aspartate transaminase (AST), alkaline phosphatase (ALP), albumin, and total bilirubin according to manufacturer instructions.

##### Real-time polymerase chain reaction (PCR)

The QIAzol Lysis Reagent (Qiagen, Cat. No. 79306) was used to isolate total RNA from the frozen liver tissue of mice according to the manufacturer’s procedure. The commercial first strand cDNA synthesis kit was used to make cDNA from 1 µg of total RNA (SensiFAST cDNA Synthesis kit (BIO-65054)). The cDNA samples are run in triplicate for real-time PCR analysis using SensiFAST SYBR Green Kit (BIO-98005), and the relative expression of genes is compared to the internal control, GAPDH. The thermal cycling protocol was as follows: initial denaturation at 95 °C for 10 min, followed by 40 cycles of amplification at 95 °C for 10 s, annealing at 66 °C for 10 s, and extension at 72 °C for 20 s. The threshold cycle (Ct) values were used to calculate the RNA concentration in each sample. The mRNA expression levels were determined using the 2^−ΔΔCT^ method in relation to the mRNA levels of the GAPDH gene. (Livak and Schmittgen 2001) The primer sequence (Sigma Aldrich) used are shown in Table 1.

**Table 1:** Primer sequence for PCR assessed parameters

Assessed RNA	Primer sequence
GAPDH	F-5′-TGCACCACCAACTGCTTAG-3′
	R-5′-GGATGCAGGGATGATGTTC-3′
MCP-1	F- 5′- CATGCTTCTGGGCCTGCTGTTC-3′
	R-5′-CCT GCT GCT GGT GAT CCT CTT GTA G-3′
TNF-α	F-5′- GCC AGC CTC CGA AGC CAG C-3′
	R-5′-GGG CGG TAG CGT CCT TGG G-3′
IL-1β	F-5′-TGG ACT TCG CAG CAC AAA ATG-3′
	R-5′-GTT CAC TTC ACG CTC TTG GAT-3′
Collagen-1	F-5'-TTCCTGCCTCAGCCACCTCA-3'
	R- 5'- GAACCTTCGCTTCCATACTCG-3'

##### Western blot

The liver tissues were homogenized on ice in RIPA buffer and centrifuged at 10,000 rpm for 30 min to separate the supernatant for western blot analysis. The protein content was determined using a BCA protein test. The samples were run on SDS-PAGE gels containing 12.5% SDS and a total protein loading volume of 40 µg per lane. The separated proteins were transferred to a PVDF membrane using a semidry transfer method (Bio-Rad, Hercules, CA, USA). The membranes were incubated with the primary antibodies; SIRT-1 (2310), α-SMA (14,968), TGF-β (3711), Iκβ (9242), p-Iκβ Ser32 (9241), and β-actin (4970) (Cell signaling technology, Beverly, MA, USA) overnight at 4 °C after blocking with 5% non-fat milk in TBST. Following primary antibody incubation, membranes were washed with TBST and then treated with anti-rabbit (1:1000) secondary antibody before being rinsed with TBST. Image lab software was used to examine the image (Bio-Rad Laboratories, Hercules, CA, USA), the intensity of each band was adjusted against β-actin levels (Burnette 1981).

#### Histopathological examination

The formalin fixed liver tissues were processed for routine paraffin block preparation. Sections of 5 μm were cut by rotatory microtome, stained with H&E stain and Masson’s trichrome stain, and examined under the light microscope for detection of granuloma formation and fibrotic changes.

### Statistical analysis

Data were tested for normal distribution by the Shapiro–Wilk test and accordingly were analyzed by one-way ANOVA followed by the Tukey’s test for multiple comparison as a Post Hoc test. Analysis was done by the GraphPad Prism 9.0.0 software and data are expressed as mean ± standard deviation (SD). Statistical significance was set at *P* < 0.05.

## Results

### Resveratrol improved portal hemodynamics

**Fig. 2:**
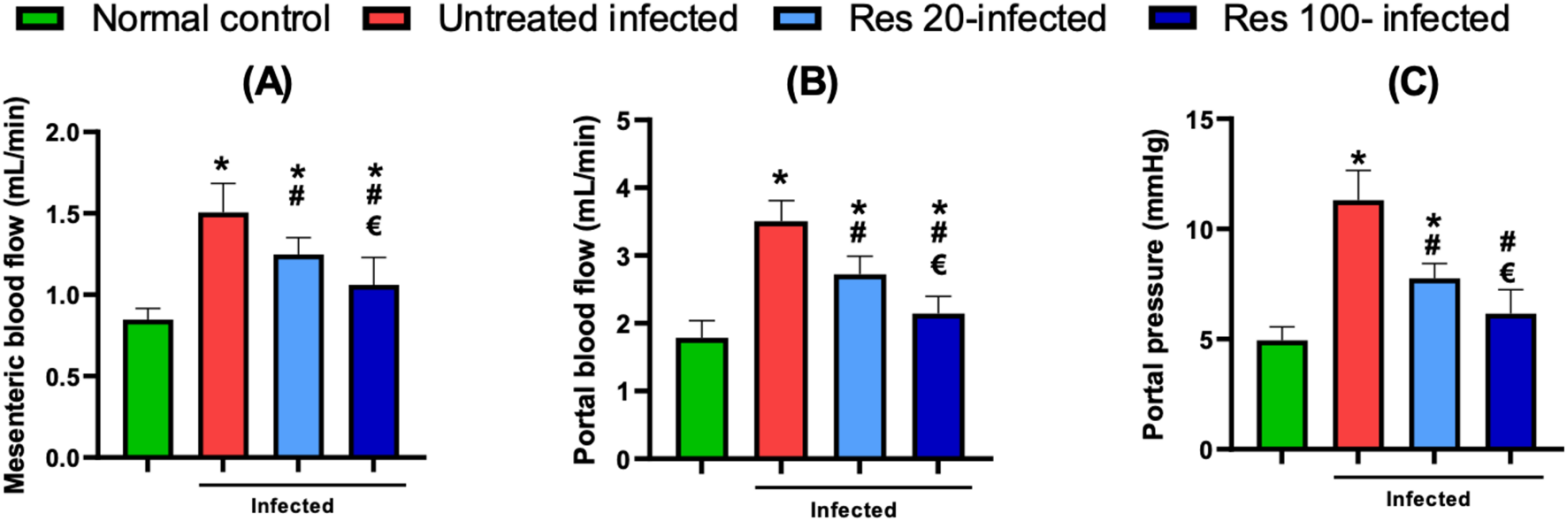
Effect of resveratrol on portal hemodynamics in *S. mansoni* infected mice. Changes in mesenteric artery blood flow are shown in (**A**), portal venous flow in (**B**), and portal pressure in (**C**)**,** Data are expressed as means ± SD. (*n* = 12) and analysed by one way ANOVA followed, by Tukey’s multiple comparisons test. *P* < 0.05 was as set as the level of significance. *significant difference versus the normal control group, **#**significant difference versus untreated infected group, and €significant difference versus the Res-20-infected mice. Res, resveratrol

Hemodynamic measurements revealed a significant increase in both the mesenteric arterial and portal venous blood flow in untreated infected mice versus the normal control. Consequently, the portal pressure was also found to be significantly elevated as well. Resveratrol-treated mice had pressure and flow values significantly lower than the untreated group. More prominent effects were seen with the higher dose of resveratrol where the portal pressure did not show a significant difference to that of the normal mice (Fig. 2).

### Resveratrol improved gross features of hepatic fibrosis and portal hypertension

**Fig. 3:**
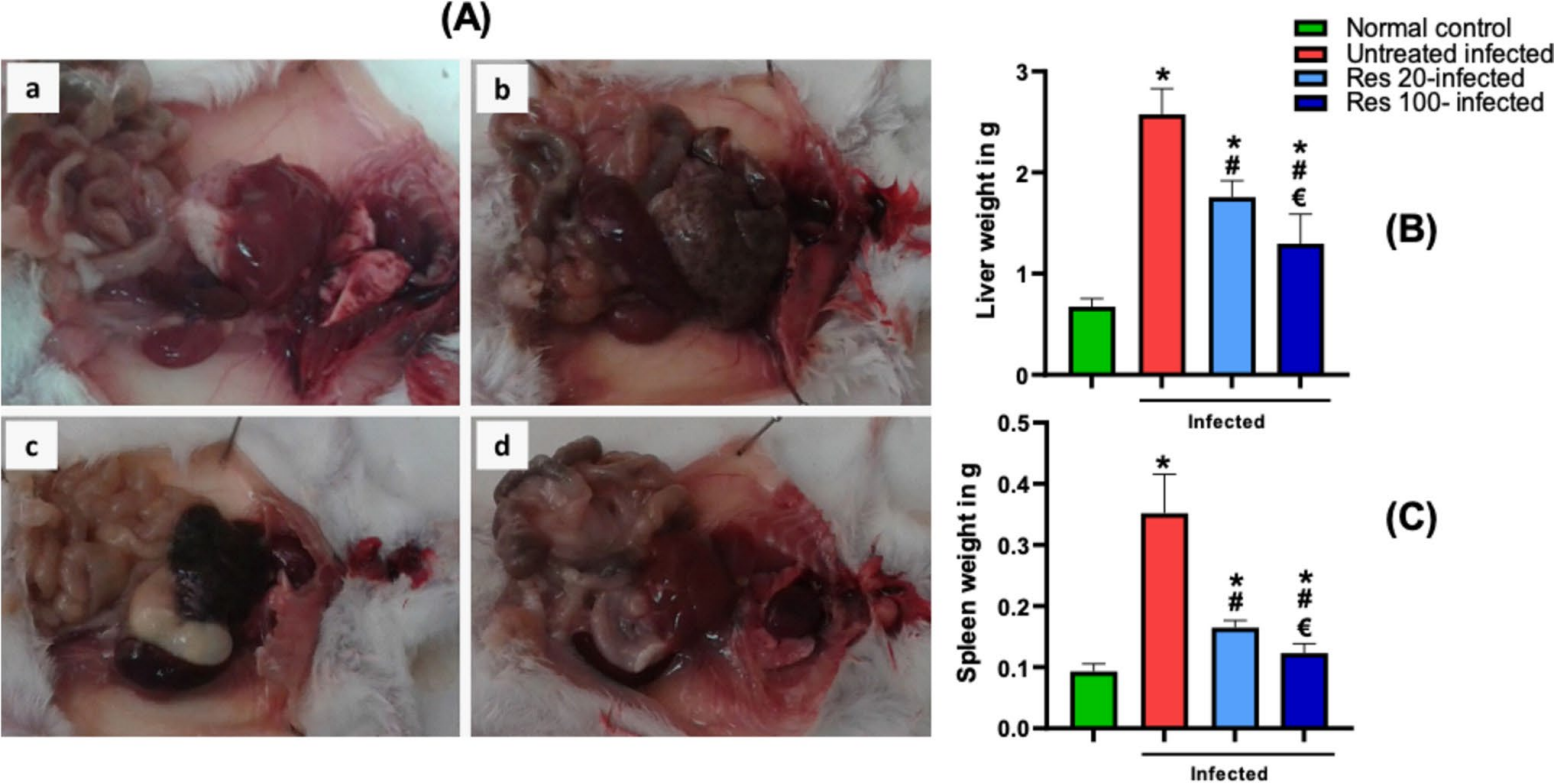
Effect of resveratrol on *S. mansoni*-induced gross pathologic changes. Representative images showing gross pathology of the liver and spleen are shown in (**A**) with an example of control mouse (**a**), infected (**b**), and infected mice treated with resveratrol in 20 and 100 mg/kg, in (**c**) and (**d**) respectively. Hepatosplenomegaly was observed in infected mice with numerous white spots seen on the surface of liver indicating granuloma nodules. The effect on absolute weights of liver and spleen is shown in (**B**) and (**C**), respectively. Data are expressed as means ± SD (*n* = 12) and analyzed by one way ANOVA followed by Tukey’s multiple comparisons test. *P* < 0.05 was as set as the level of significance. *significant difference versus the normal control group, **#**significant difference versus untreated infected group, and €significant difference versus the Res-20-infected mice. Res, resveratrol

Examination of both the liver and spleen at 10 weeks after *S. mansoni* infection showed evident gross hepatic fibrotic changes with extensive granuloma formation as shown by the nodular surface appearance, white spotting, and firm consistency. This was associated with a significant hepatosplenomegaly compared with the normal control. Resveratrol treatment significantly reduced the absolute weight of both the liver and spleen and mitigated the gross fibrotic changes in a dose-dependent manner (Fig. 3).

### Resveratrol ameliorated *S. mansoni*-induced histopathological changes

**Fig. 4:**
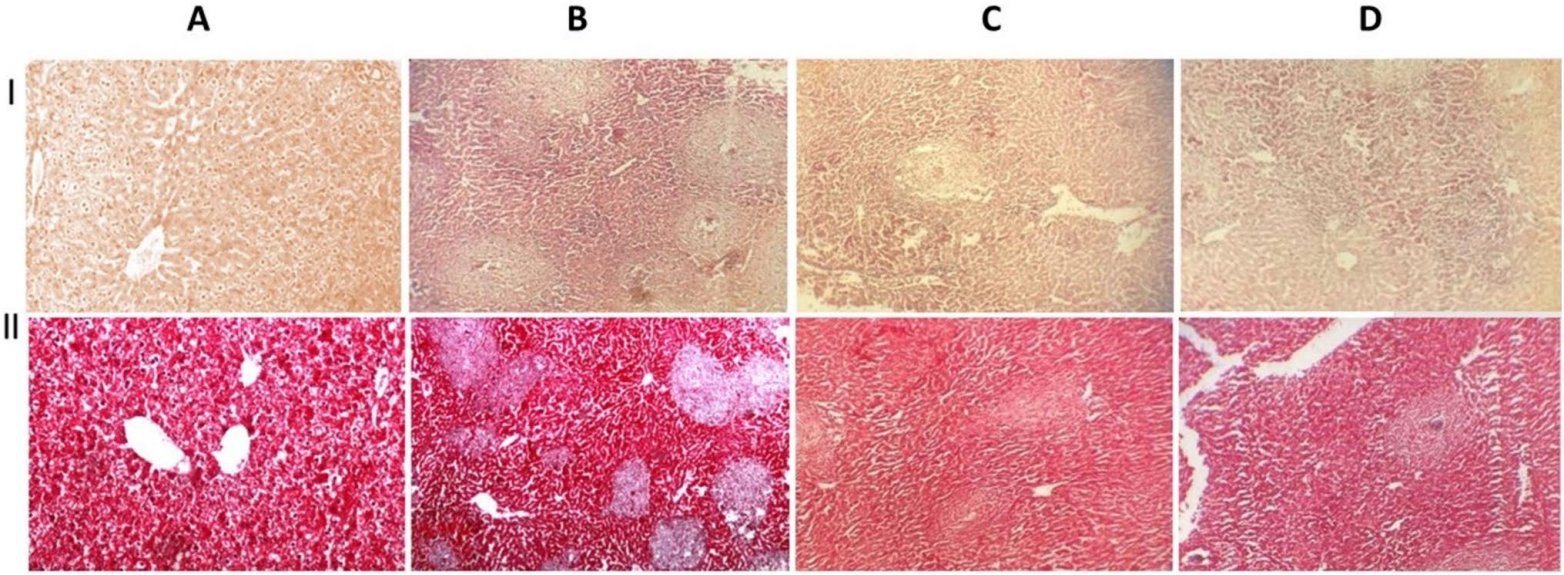
Representative photomicrographs of light microscopic picture of H&E and Masson’s trichrome stained hepatic tissue sections in I and II, respectively (Mic. Mag × 100). **A** represents liver tissue sections obtained from normal control mice showing normal liver architecture with polyhedral acidophilic hepatocytes and vesicular rounded central nuclei, radiating from the central vein. Blood sinusoids were seen in between cords of hepatocytes with almost negative staining with the Masson’s trichrome stain. Multiple granulomas and periportal fibrotic changes with intense inflammatory infiltrate are seen in tissue sections obtained from untreated *S. mansoni-*infected mouse (**B**). Collagen fibers are highlighted in blue by the Masson’s trichrome stain (**C**) represents hepatic sections of infected mice treated with 20 mg/kg of resveratrol, with only mild periportal fibrotic changes and mild to moderated lymphocytic infiltration. Almost normal liver architecture with mild inflammatory infiltrate and minimal periportal fibrosis are shown in liver sections of mice from the 100 mg/kg resveratrol-treated group (**D**)

Histopathological examination of tissue sections obtained from *S. mansoni*-infected mice showed numerous bilharzial granulomata with inter-granulomatous fibrosis and portal fibrosis as highlighted by Masson’s trichrome stain. Almost all of Res-20 treated mice showed few granulomas with mild to moderate inflammatory infiltrates mainly as lymphocytes seen in portal tracts and sinusoids. Mild fibrosis was seen in portal tracts as shown in Masson’s trichrome-stained sections. Treatment with the high dose of resveratrol was associated with similar findings, but the inflammatory changes were mild with no observed granulomas, while Masson’s trichrome stain showed minimal to absent fibrotic changes (Fig. 4).

### Resveratrol improved serum markers of hepatic functions

**Fig. 5:**
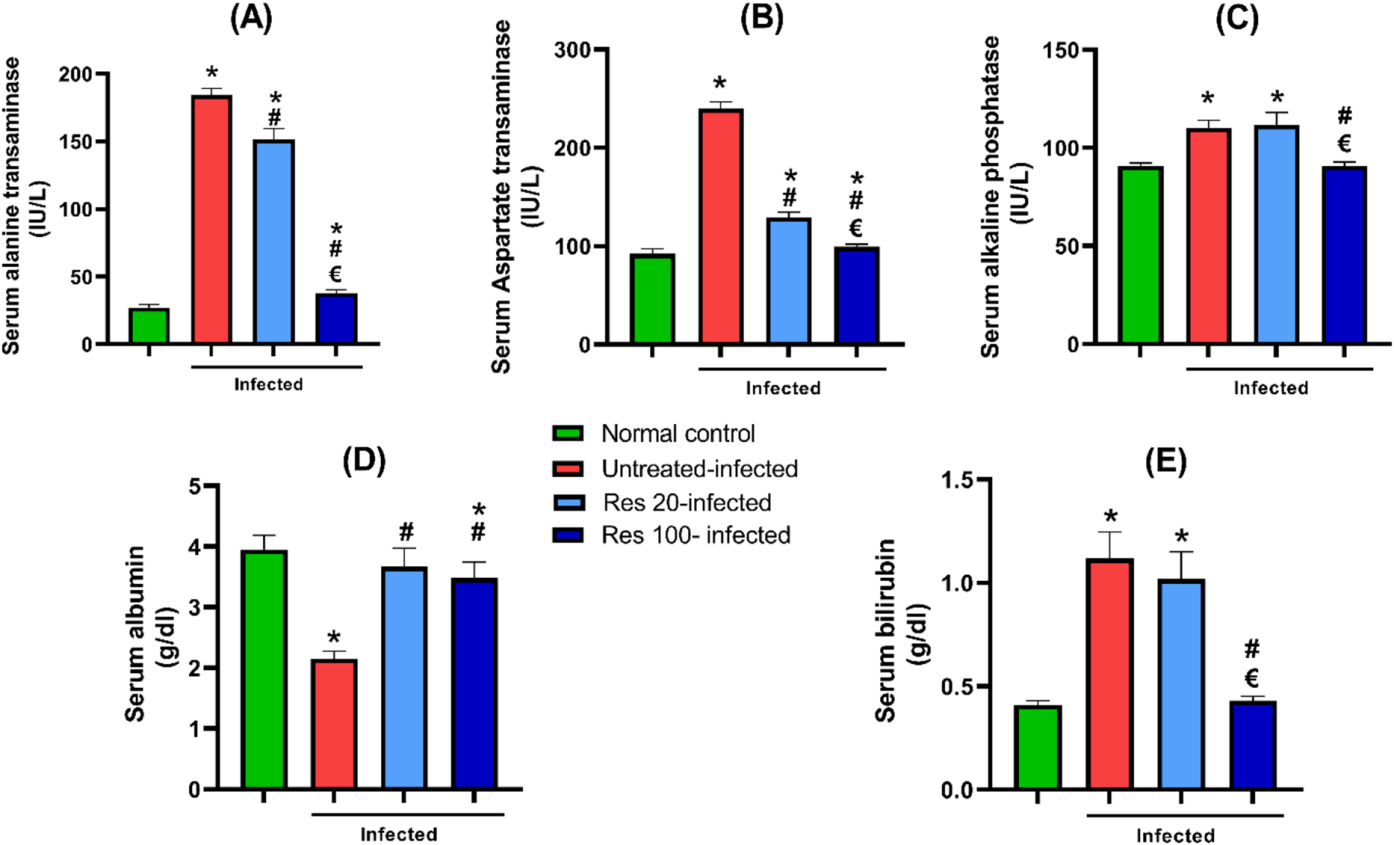
Effect of resveratrol on serum markers of hepatic function in *S. mansoni* infected mice. Changes in serum alanine aminotransferase are shown in (**A**), aspartate aminotransferase in (**B**), alkaline phosphatase in (**C**)**,** albumin in (**D**) and bilirubin in (**E**). Data are expressed as means ± SD. (*n* = 12) and analyzed by one-way ANOVA followed by Tukey’s multiple comparisons test. *P* < 0.05 was as set as the level of significance. *significant difference versus the normal control group, **#**significant difference versus untreated infected group, and €significant difference versus the Res-20-infected mice. Res, resveratrol

A significant elevation of the liver enzymes reflecting hepatic cell injury was detected in the serum of mice of untreated infected group. This was associated also with significantly lower levels of serum albumin and increased bilirubin. Treatment with resveratrol in both doses was associated with a significant decrease in ALT, AST, and an increase in albumin versus the untreated mice, while only the higher dose ameliorated the increase in ALP and bilirubin levels (Fig. 5).

### Resveratrol reduced hepatic TNF-α, IL-1β, MCP-1 and collagen 1 gene expression

**Fig. 6:**
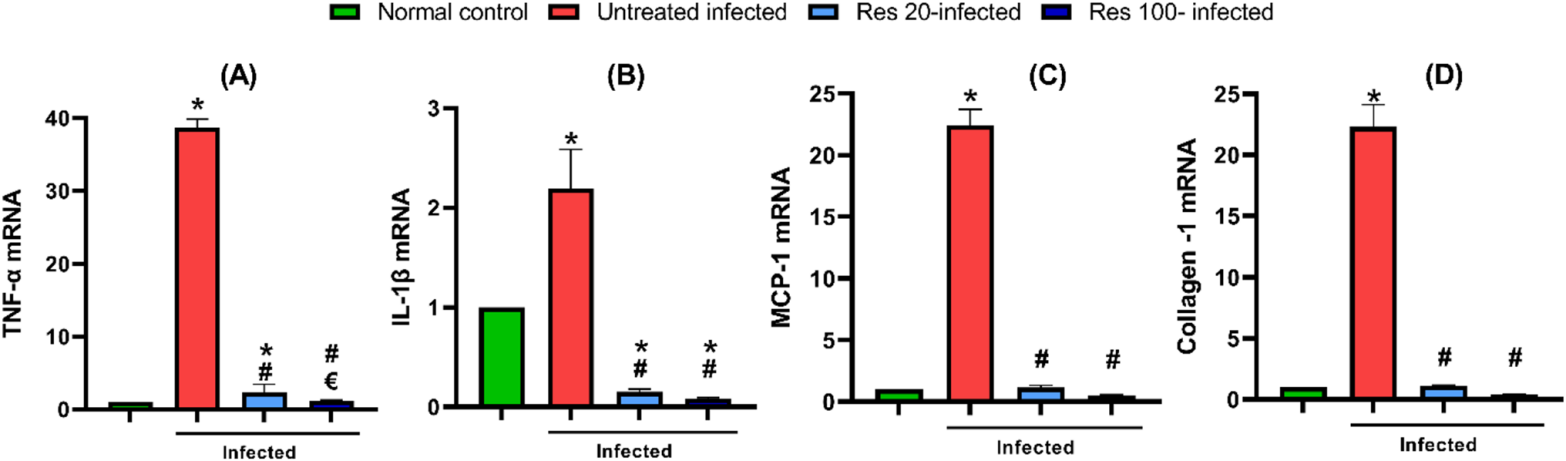
Effect of resveratrol on hepatic inflammatory and fibrotic markers gene expression in *S. mansoni* infected mice. The expressions of TNF-α (**A**), IL-1β (**B**), MCP-1 (**C**) and collagen-1 (**D**) mRNA were detected by quantitative real-time RT-PCR. The mRNA expression levels were normalized to the housekeeping gene (GAPDH) and expressed as fold change. Data are expressed as means ± SD. (*n* = 12) and analysed by one way ANOVA followed by Tukey’s multiple comparisons test. *P* < 0.05 was as set as the level of significance. *significant difference versus the normal control group, **#**significant difference versus untreated infected group, and €significant difference versus the Res-20-infected mice. Res, resveratrol; TNF-α, tumor necrosis factor-alpha; IL-1β, Interleukin 1beta; MCP-1, chemoattractant protein-1

Analysis of hepatic tissue mRNA of *S. mansoni*-infected mice revealed induction of expression of inflammatory cytokines and chemokines genes as shown by the significantly increased TNF-α, IL-1β and MCP-1 mRNA together with upregulated collagen-1 as a marker of fibrosis. Resveratrol treatment almost normalized the elevated markers, with a remarkable suppressive effect on IL-1β, that was even significantly lower than the normal control (Fig. 6).

### Resveratrol reduced hepatic TGF-β1, α-SMA, suppressed NF-κB pathway and upregulated Sirt-1

**Fig. 7:**
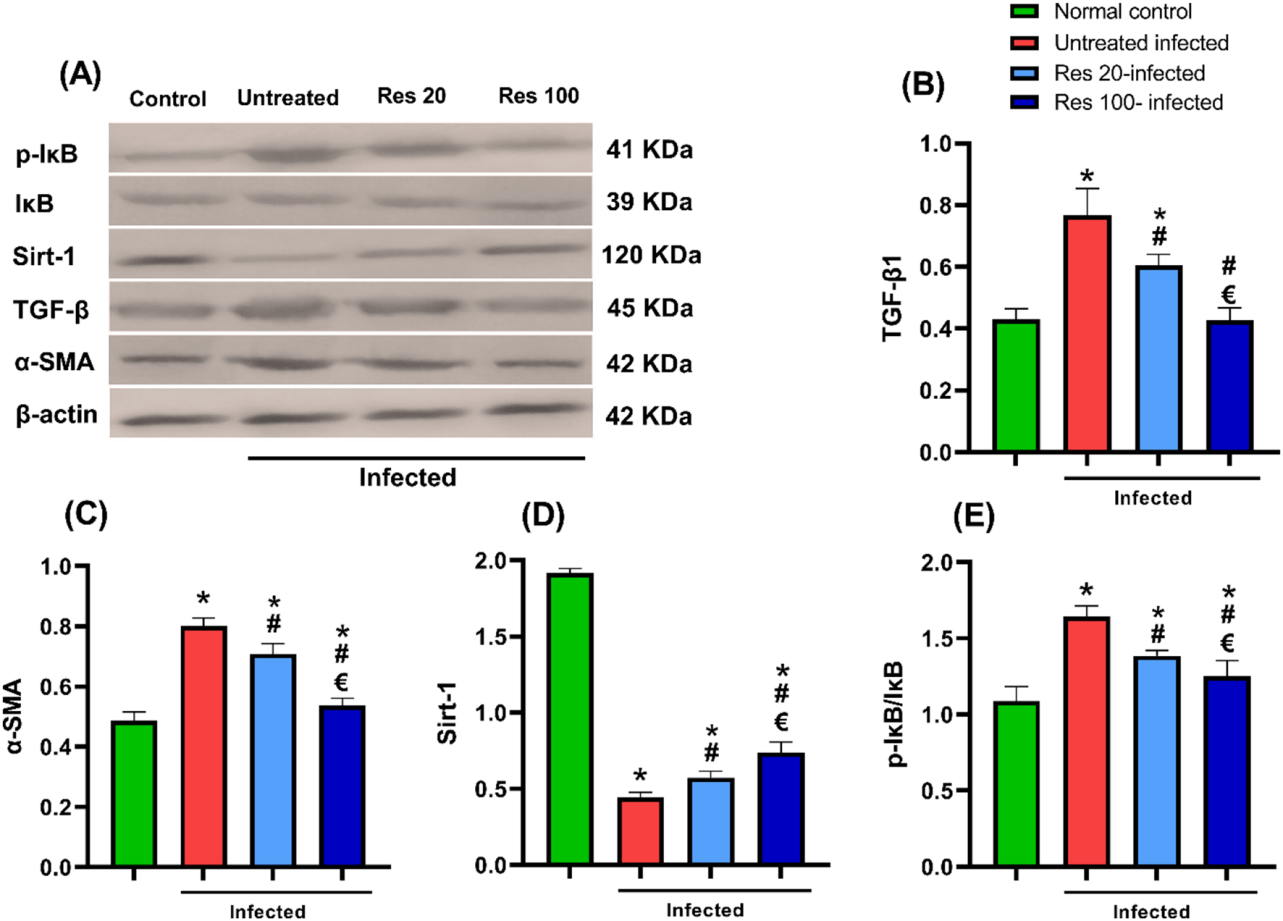
Effect of resveratrol on hepatic TGF-β1, α-SMA, Sirt-1 and IκB activity assessed by western blotting. Representative immunoblots of TGF-β1, α-SMA, Sirt-1, IκB, p-IκB and β-actin proteins are shown in (**A**). The level of protein expression was normalized to that of β-actin and the relative expressions of TGF-β1 (B), α-SMA (**C**), and Sirt-1 (**D**) were represented. The ratio of phosphorylated to unphosphorylated Iβκ was calculated and presented in (E). Data are expressed as means ± SD (*n* = 12) and analysed by one way ANOVA followed by Tukey’s multiple comparisons test. *P* < 0.05 was as set as the level of significance. *significant difference versus the normal control group, **#**significant difference versus untreated infected group, and €significant difference versus the Res-20-infected mice. Res, resveratrol; TGF-β1, tissue growth factor beta-1; α-SMA, alpha smooth muscle actin; Sirt-1rtuin 1, and IκB, inhibitor kappa B

Western blot analysis of hepatic tissue homogenate showed significant increased expression of the measured fibrotic markers proteins of TGF-β1 and α-SMA in *S. mansoni*-infected mice versus normal mice. Infection was also associated with significantly elevated p-IκB/IκB ratio. Treatment of resveratrol dose dependently reduced the upregulated markers. Conversely, a significant reduction of Sirt-1 was detected in the untreated infected animals and this reduction was partially mitigated in resveratrol-treated mice (Fig. 7).

## Discussion

Schistosomiasis is the second most serious transmissible disease owing to its great impact on infected subject morbidity and mortality (Colley et al. 2017). The main complication of schistosomiasis as variceal bleeding secondary to liver fibrosis and portal hypertension is crippling and yet, life threatening. Therefore, prevention of fibrosis remains the only feasible strategy available (Kong et al. 2020). Though very few agents (e.g., pirfenidone and nintedanib) have recently been approved for pulmonary fibrosis, an effective antifibrotic drug that can be used safely in liver fibrosis is considered a big challenge (Fang et al. 2021). Therefore, promising plant-derived compounds possessing pleiotropic pharmacological actions with a long history of safety, such as resveratrol, merits attention (Koushki et al. 2018).

In this study, we document an evident antifibrotic effect of resveratrol in schistosomal hepatic fibrosis and delineate some contributing mechanisms. In accordance with previous studies (Fang et al. 2021) (El-Lakkany et al. 2012), the untreated *S. mansoni* infected mice showed an elevation in serum levels of liver enzymes reflecting the loss of hepatocyte integrity, whereas the detected hypoalbuminemia could be the result of decreased albumin synthesis from damaged hepatocytes or due to decreased absorption from damaged intestinal mucosa (Al Humayed et al. 2020; El-Lakkany et al. 2012).

Hepatocyte damage occurs secondary to the depicted massive hepatic proinflammatory state evidenced by the significant elevated expression of the inflammatory cytokines and chemokine genes with subsequent stimulation of the fibrotic process. In fact, HSCs are recognized to serve as the primary target for many inflammatory stimuli of fibrogenesis (Hou and Syn 2018; Seki and Schwabe 2015). It is thus expected that suppression of the release of inflammatory mediators from injured hepatocytes and Kupffer cells is crucial for mitigating HSCs activation and consequently suppression of fibrogenesis (El-Agamy et al. 2011).

Additionally, the observed upregulation of TGF-β1, α-SMA and ultimately collagen 1 coincides with the demonstrated histological findings of *S. mansoni*-induced liver injury. TGF-β1 is a pleiotropic cytokine that displays proinflammatory, fibrogenesis, and immunosuppressive properties (Mantel and Schmidt-Weber 2011). In the context of liver schistosomiasis, TGF-β1 expression has been demonstrated to be increased in liver tissues from animal models as well as patients with liver schistosomiasis (Chen et al. 2008; El-Lakkany et al. 2011, 2012; Li et al. 2014a, b). It plays a role in activation and differentiation of HSCs into myofibroblast phenotype that expresses α-SMA and deposits excessive amount of extracellular matrix, predominantly collagen type I (Gressner and Weiskirchen 2006). In turn, the activated HSCs stimulate TGF-β, and a vicious circle ensues (Wahl et al. 1997). The critical role of TGF-β1 was further emphasized by the ability of TGF-β1 soluble receptors (Yata et al. 2002) or TGF-β neutralizing antibody to scavenge TGF-β1 and reverse hepatic fibrosis in experimental animals (Ling et al. 2013). Nevertheless, a substantial body of evidence highlights other pathways contributing to schistosomal fibrosis which either minimally involves TGF-β1 or is completely TGF-β1 independent (Kaviratne et al. 2004; Liu et al. 2014).

Herein, we detected a significant increase in phosphorylation of the Iκβ which triggers its recognition by an E3 ligase, its polyubiquitination and subsequent degradation by the proteasome. Such degradation will release and thus switch on the translocation of NF-κB, the master key of the inflammatory response to the nucleus activating cytokines, chemokines, and immunoreceptors gene expression in addition to the profibrogenic cytokine TGF-β. The functional nature of NF-κB target genes underpins the harmful consequences of its dysregulated activity and necessitates its tight control (Christian et al. 2016). Accumulating data points to the essential role of NF-κB in promoting HSCs activation and survival, and engendering fibrosis (Marcher et al. 2019; Muriel 2009). In this context, an increase in NF-κB binding activity in liver tissues from Schistosoma japonicum infected mice was previously reported confirming its role in mediating hepatic schistosomiasis (Chen et al. 2008).

The complex interaction of the different pathways contributing to hepatic fibrosis would finally lead to portal hypertension associated with splanchnic vasodilation (Abraldes et al. 2006). Consistent with previous studies reporting the development of a state of hyperdynamic circulation in human as well as animal models of portal hypertension (Bolognesi et al. 2014; Sarin et al. 1991; Strauss 2002), we detected a significantly elevated portal pressure associated with a significant increase in the mesenteric blood flow when measured 10 weeks post cercarial exposure. Clinically wise, splenomegaly, as detected here, is an important manifestation of abnormally raised portal pressure (Kong et al. 2020). It has been suggested that splenomegaly plays a role in the progression of liver fibrosis to cirrhosis. Although the precise mechanism has not been fully elucidated, the excessive production of TGF-β1 from splenic macrophages could be a critical contributing factor (Li et al. 2017).

Although resveratrol has gained much interest in the management of liver diseases (Bishayee et al. 2010), its effect on portal pressure in *S. mansoni*-infected mice was not addressed in previous works. Our results showed a significant decrease in portal pressure, portal flow, spleen weight by both the low and high doses of resveratrol.

The pathophysiologic mechanisms of portal hypertension involve increased flow in portal vessels and/or increased intrahepatic vascular resistance (Gunarathne et al. 2020), The former occurs as a consequence of splanchnic vasodilation whereas the latter reflects hepatic architectural changes involving myofibroblasts, HSCs, and vascular smooth muscle cells (VSMCs) which play a central role in regulating the intrahepatic vascular tone. Moreover, dysfunction of sinusoidal endothelium with decreased nitric oxide (NO) production or function greatly affects the tone of the adjacent VSMCs (Mehta et al. 2014). The net result is increased pressure in the portal circulation due to an increase in blood flow.

Treatment with resveratrol produced a reduction in portal pressure that was more prominent with the higher dose. In line, Di Pascoli et al. (2013), reported a favorable effect of resveratrol on portal pressure in carbon tetrachloride (CCl_4_)-induced cirrhotic rats. This beneficial effect could be ascribed to inhibition of HSC activation secondary to the observed dose-dependent suppression of TGF-β and α-SMA gene expression; both have strong stimulatory effect on HSCs. This suppression was associated with consequent reduction in the expression of collagen I and preservation of liver architecture. Indeed, the gross appearance of the liver tissue obtained from the resveratrol-treated mice was significantly improved especially with the higher dose. This was reflected microscopically on near total mitigation of fibrotic changes, decreased granulomas, and the distinct resolution of inflammatory infiltrations.

In concordance, favorable effects of resveratrol on various models of liver injury have been demonstrated in both in vitro and in vivo studies (Bishayee et al. 2010; Muriel and Rivera-Espinoza 2008). It exerts protective effect against various hepatotoxicants (Al Humayed et al. 2020; Bingul et al. 2021; Lee et al. 2010). Likewise, resveratrol exhibited a beneficial effect against liver diseases such as chronic biliary obstruction (Ara et al. 2005), and non-alcoholic fatty liver disease (Ara et al. 2005; Li et al. 2014a, b).

A previous study examined the effect of resveratrol, among others, as preventive and therapeutic agents against liver fibrosis induced by *Schistosoma mansoni* infection. Noticeably, they found that resveratrol can prevent the development of liver fibrosis, but it had no effect on reversal of fibrosis (El-Agamy et al. 2011).

Although there has been tremendous progress over the past decade in understanding the antifibrotic effect of resveratrol, the detailed molecular mechanisms remain elusive (Izzo et al. 2021). For instance, resveratrol is well recognized to exert effective antioxidant effects owing to its capability to promote the synthesis and improve the activity of antioxidant molecules. Nevertheless, it failed to prevent markers of oxidative stress in a model of CCl_4_-induced hepatic injury (Pan et al. 2017). Hence, it is assumed that the anti-fibrotic effect of resveratrol is independent of the sole antioxidant effect.

Several studies reported that natural compounds including resveratrol mitigates various pathological conditions via interfering with NF-κB signaling pathway (Laurindo et al. 2023; Ren et al. 2013; Xu et al. 2018). As formerly discussed, we detected a significant increase in the p-IκB/IκB ratio in the untreated-infected mice which triggers the subsequent release of NF-κB and the enhanced transcription of its target genes as reflected by the increased cytokines and chemokine. However, to date, the effect of resveratrol on translocation, DNA-binding, and phosphorylation of the key components in NF-κB signaling pathway is controversial (Ren et al. 2013; Tsai et al.^1999^). For instance, Chavez et al. (2008) demonstrated that the antifibrotic effect of resveratrol in chronic CCl_4_-induced liver was associated with a non-significant decrease in the relative optical density of nuclear NF-κB. It is worth noting that they employed a single dose of resveratrol in their study (10 mg kg/kg orally, for 8 weeks), therefore, a positive effect of a higher dose cannot be ruled out.

On the other hand, an earlier study reported that resveratrol inhibited the nuclear translocation of NF-κB but it showed no effect on the phosphorylation nor degradation of IκBα (Manna et al. 2000). On the contrary, Pellegatta et al. demonstrated that a longer period of incubation with resveratrol could inhibit phosphorylation of NF-κB components and IκBα in human endothelial cells (Pellegatta et al. 2003).

In fact, our finding revealed a dose-dependent inhibition of IκB activity as shown by the significant decrease in the p-IκB/IκB ratio with both doses of resveratrol versus the untreated mice. In line with these data, Ren et al. precluded any effect of resveratrol on DNA-binding activity and transportation of NF-κB. Rather, it blocked the activity, not the expression, of IKBα. Additionally, resveratrol inhibited IκB kinases (IκK) activity that catalyzes the phosphorylation of Iκβ, to further inhibit the canonical activities of NFκB in a dose-dependent manner (Ren et al. 2013).

Remarkably, it has been speculated that resveratrol should act upon upstream molecules to inhibit NF-κB activation by various stimuli. Indeed, data suggest that pharmacological activation of SIRT1 by resveratrol could be a crucial underlying mechanism of its anti-inflammatory, antiproliferative, and antiapoptotic effects (Iside et al. 2020).

Intriguingly, we detected a significantly reduced SIRT1 in the untreated infected mice, which may point to its implication in the pathological consequences of schistosomal infection including fibrosis. Of note, there is a number of critical proofs documenting the protective role of SIRT1 against fibrosis as it controls Smad fibrotic pathway in HSCs. Further, reduced SIRT1 expression or HSC-specific deletion of SIRT1 led to exacerbated fibrosis in mice models (Li et al. 2018; Ren et al. 2022). In line with our data, SIRT1 protein expression was also decreased in liver infected by *S. japonicum* (Zhou et al. 2021).

SIRT1 has been reported to participate in various physiological and pathological states by controlling expression and activities of several regulatory molecules. Previous studies showed that the protective effect of resveratrol is linked to an increase in SIRT1 activity. For instance, Yu et al. showed that resveratrol reduced the inflammatory response induced by partial liver resection through reduction of high mobility group box 1 (HMGB1) translocation to the nucleus as a result of SIRT1 upregulation (Yu et al. 2019). Furthermore, SIRT1 regulates the expression of antioxidant enzymes, and this effect is implicated for the antioxidant effect of resveratrol (Pan et al. 2017).

Interestingly, an antagonistic crosstalk between NF-κB and SIRT1 signaling pathways in regulating immune and inflammatory response to infection has been uncovered (Kauppinen et al. 2013). This antagonism has double benefits. On one hand, the body needs to respond quickly to harmful stimuli such as an infection or tissue damage by switching to a rapid energy generation system through suppressing SIRT1. On the other hand, it is necessary to switch off the pro-inflammatory milieu once the harmful stimulus has disappeared. The disturbance of this antagonistic balance with sustained stimulation of NF-κB on the expense of active SIRT1 is the centerpiece in chronic liver disease (de Gregorio et al. 2020). Indeed, The HDAC activity of SIRT is the key for its antagonistic effect on NF-kB. Deacetylation of RelA/p65 subunit of NF-κB, impairs its transcriptional activity and further predisposes to its ubiquitination and degradation. The deacetylation of RelA/p65 by SIRT1 also favors the re-association of NF-κB with IκB-α which triggers the back transport of the NF-κB complex from the nucleus to the cytoplasm regaining the inactive state (Rothgiesser et al. 2010; Yang et al. 2010). Thus, the activation of SIRT1 by resveratrol in our study would further explain the suppressed transcription of NF-κB downstream genes, including those encoding IL-1β, TNF-α, TGF- β and MCP-1, and other pro-inflammatory factors (Meng et al. 2021; Tian et al. 2016).

Finally, another debatable point is whether PZQ exerts direct antifibrotic effect aside from its schistosomicidal effect. Previous studies have demonstrated that praziquantel reduced fibrosis as a result of amelioration of the immune and inflammatory responses in different mice models of liver injury (El-Lakkany et al. 2012; Liang et al. 2011; Liu et al. 2014; Singh et al. 2019). Conversely, PZQ was recently reported to have a limited potential for prevention and/or reversal of the already established fibrosis (Nono et al. 2020). In our study, PZQ was administered to both the resveratrol-treated and untreated groups to rule out any significant beneficial effect of praziquantel alone in attenuating fibrosis.

In conclusion, the present study demonstrates that resveratrol could protect against the deleterious hemodynamic and structural consequences of *S mansoni*-induced liver injury. The beneficial effects of resveratrol can be mainly attributed to its suppressive effect on NF-κB signaling and its downstream proinflammatory and fibrotic transcriptional products. The evidence on the ability of resveratrol to restore the distorted balance of the SIRT1/NF-κB axis, encourages its further clinical evaluation as a promising agent in schistosomal hepatic fibrosis.

## Data Availability

All data generated or analyzed during this study are available from the corresponding author on reasonable request.

## Abbreviations

ALP: Alkaline phosphatase
ALT: Alanine transaminase
AST: Aspartate transaminase
HDACs: Histone deacetylases
IL-1β: Interleukin 1beta
IκB: Inhibitory kappa B
MCP-1: Chemoattractant protein-1
NF-κB: Nuclear factor kappa B
NO: Nitric oxide
PZQ: Praziquantel
Res: Resveratrol
SEA: Soluble egg-antigens
SIRT1: Silent information regulator 1
TNF-α: Tumor necrosis factor-alpha
VSMCs: Vascular smooth muscle cells
